# A Comprehensive Bibliometric Analysis of the Sport of Squash (1973–2024): Progress, Collaboration, Findings, and Thematic Evolution

**DOI:** 10.3390/sports13060157

**Published:** 2025-05-23

**Authors:** Ruizhi Liu, Miran Kondrič, Jihong Wang

**Affiliations:** 1 China Table Tennis College, Shanghai University of Sport, Shanghai 200438, China; liuruizhi@sus.edu.cn; 2 Faculty of Sport, University of Ljubljana, 1000 Ljubljana, Slovenia; miran.kondric@fsp.uni-lj.si; 3 The School of Athletic Performance, Shanghai University of Sport, Shanghai 200438, China

**Keywords:** squash, athletic performance, health promotion, bibliometric analysis, racket sports

## Abstract

Squash, which originated in the early 19th century, was officially incorporated into the 2028 Los Angeles Olympic Games by the International Olympic Committee in 2023. This inclusion marks a significant milestone, highlighting its growing international recognition and potential for global development. However, squash has historically been underrepresented in academic research compared to other popular sports, with studies being scattered and relatively underdeveloped. To systematically evaluate the current state of squash research and foster academic development in the field, this study employs a combination of bibliometric and qualitative analyses, aiming to provide a comprehensive overview of squash research in terms of research progress, collaboration networks, key findings, and thematic evolution. Drawing on Web of Science, Scopus, and PubMed data, the analysis covers 206 publications from 36 countries spanning from 1973 to 2024. Network analysis based on co-authorship frequency and geographic clustering identifies the UK and Australia as leading contributors, with extensive collaboration among institutions and authors. Performance analysis (tactical and technical modeling), physiological profiling (energy demands), and medical studies (injury epidemiology) emerged as the three core research areas. The study reveals a shift in research focus from safety and physiological characteristics to performance enhancement, which is driven by technological innovation. Future research should prioritize underdeveloped areas such as youth development, psychology, and nutrition while integrating diverse participant groups (e.g., female athletes, recreational players) and advanced technologies.

## 1. Introduction

Squash, originating in the early 19th century at Harrow School in England, has undergone significant development over the past two centuries. Recognized as “the healthiest sport” [1], squash has seen rapidly expanding participation among juniors, adults, women, middle school, high school, and college students. More than 20 million enthusiasts currently engage in squash across 185 countries worldwide [2]. In 2023, the International Olympic Committee (IOC) officially included squash in the 2028 Los Angeles Olympic Games, highlighting its growing international recognition and potential for global development.

Modern squash is a fast-paced, high-intensity sport played in an enclosed court. Players take turns hitting the ball against the front wall, aiming to prevent their opponents from making a valid return and thereby scoring a point [3]. This sport requires a high level of physical fitness and incorporates elements of tactics, psychology, sports injuries, and injury prevention, making it a complex, multidisciplinary sport [4]. However, compared to collective sports like basketball (4496 publications in 2000–2023) [5] and football (7243 publications in 2010–2023) [6] or other racket sports such as tennis (733 publications until 2017) [7], badminton (856 publications in 1939–2020) [8], and table tennis (35,308 publications in 1963–2018) [9], squash has received significantly less academic attention. Current research focuses on performance-related aspects such as physical fitness, physiology, match analysis, and injury prevention [10]. Existing reviews primarily provide systematic summaries of these topics [4,11], yet they are often outdated and lack cross-sectional comparisons among different research areas.

While foundational in academic research, traditional literature reviews have specific shortcomings, such as a lack of systematic approach, susceptibility to bias, and incomplete coverage of studies, which can limit their effectiveness and reliability [12]. In the context of the rapid accumulation of scientific research, relying solely on traditional literature reviews makes it difficult for the academic community to fully identify the development trend and current state of squash research. In contrast, bibliometric analysis quantitatively examines large volumes of literature, offering an objective view of patterns, trends, and impacts within a specific research field [13]. By employing techniques such as co-occurrence analysis, author collaboration networks, and co-citation analysis, researchers can capture research frontiers with precision and reveal intrinsic connections within knowledge structures, thereby providing data-driven support and references for decision-making in disciplinary development. This method has become a trend in academic research in recent years [14].

Therefore, this study adopts a combination of bibliometric and qualitative analyses. By constructing a knowledge map of squash research, this study systematically examines research progress, research collaborations, key findings, and thematic evolution in this field, aiming to provide a systematic mapping of the academic research landscape of this sport. Specifically, this study seeks to answer the following questions:

1. What is the current research progress in squash? How many publications exist, what areas do they cover, and in which journals are they primarily published?
2. Where are the main research forces in squash located? Which countries, institutions, and authors are actively conducting the related research?
3. What are the main components of the knowledge structure of squash research, and which publications are considered influential?
4. What are the current research hotspots in squash? How have they developed and evolved, and what is their current state?

By addressing these questions, this study aims to provide systematic reference for future researchers and establish a theoretical foundation for squash’s scientific development.

## 2. Methods

### 2.1. Bibliometric Analysis

As previously mentioned, bibliometric analysis is a method for systematically studying scientific data through statistical and analytical techniques to identify patterns, trends, and impacts in a specific field [13]. The process typically includes collecting data from relevant databases, cleaning and refining the data, and applying various bibliometric methods to generate meaningful maps and insights. This approach is essential in quantifying research output and impact within a field [15]. The knowledge maps and bibliographic information were obtained using visualization software. By integrating in-depth literature analysis with quantitative and qualitative approaches, this study aims to offer a comprehensive overview of the current research landscape of squash.

### 2.2. Data Sources and Search Strategy

This study selected Web of Science (Core Collection: Science Citation Index Expanded and Social Sciences Citation Index), Scopus, and PubMed as data sources due to their broad multidisciplinary coverage and rigorous indexing standards. Two authors (R.L. and J.W.) developed the search strategy. Keywords and Boolean operators were iteratively tested and refined to ensure the comprehensiveness and disciplinary relevance of the search scope. Another co-author (M.K.) independently validated the final query syntax against inclusion criteria.

Search terms were designed to encompass variations of the term “squash” in the context of sports, explicitly excluding agricultural terms (e.g., cucurbita, pumpkin). The document types were limited to articles and review articles published in English, with no restrictions on publication date. Data retrieval was completed on December 31, 2024. The specific search strategy is as follows:

- Web of Science: TS=(squash AND (sport* OR game* OR match* OR play*) NOT (agriculture OR plant* OR vegetable)) AND (DT=Article OR DT=Review) AND LA=English
- Scopus: (TITLE-ABS-KEY (squash AND (sport* OR game* OR match* OR play*)) AND NOT TITLE-ABS-KEY (cucurbita* OR pumpkin OR vegetable)) AND (LIMIT-TO (LANGUAGE, “English”)) AND (LIMIT-TO (DOCTYPE, “ar”) OR LIMIT-TO (DOCTYPE, “re”))
- PubMed: (((squash [Title/Abstract]) AND (sport*[Title/Abstract] OR game*[Title/Abstract] OR match*[Title/Abstract] OR play*[Title/Abstract]))) NOT (cucurbita* OR pumpkin OR vegetable*[MeSH Terms]) Filters: Full text, English

### 2.3. Literature Screening

**Figure 1:**
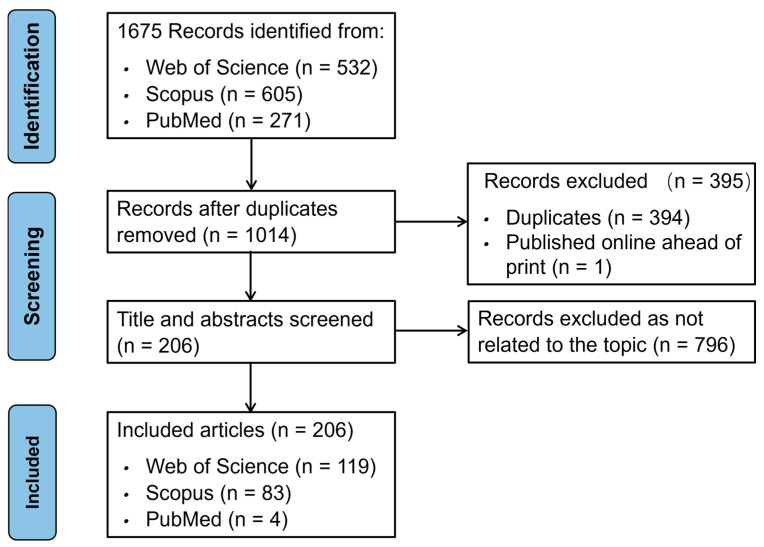
PRISMA screening flowchart.

The literature screening process adhered to the PRISMA guidelines [16], as illustrated in Figure 1. After data export from the selected databases, duplicate entries were removed using EndNote20.6. Priority was given to records from Web of Science due to its comprehensive metadata, with Scopus and PubMed following. Given the multiple meanings of “squash” in agriculture, medicine, and computer science, additional manual screening based on titles and abstracts was conducted following de-duplication by EndNote. Publications meeting any of the following criteria were excluded:

- The document type is not an article or review article.
- Duplicate publications.
- The research content is unrelated to squash as a sport.
- Research focusing on common characteristics of athletes across multiple sports will be excluded if the number of sports studied exceeds four, even if squash players are included.

The initial search yielded 1675 records: 532 from Web of Science, 605 from Scopus, and 271 from PubMed. After removing 394 duplicates and one preprint article, 1014 unique publications remained for screening.

### 2.4. Data Cleaning and Analysis Tools

Data cleaning was conducted to refine the bibliographic dataset, ensuring accuracy and consistency in analysis. VOSviewer (version 1.6.20) and CiteSpace (version 6.3.R1) were employed for bibliometric visualization and network analysis. VOSviewer facilitated network construction and clustering analysis, while CiteSpace was used for citation burst detection and temporal mapping. The cleaning process included the following:

- Standardizing the names of countries, institutions, authors, journals, and references to ensure consistency. For instance, “England”, “Scotland”, and “Wales” were consolidated into “United Kingdom”. “University of New South Wales” and “UNSW Sydney” were unified as “UNSW Sydney”. Variations of author names like “M.D. Hughes”, “Mike D. Hughes”, and “Mike Hughes” were standardized as “Mike Hughes”. Journal names, such as “Journal of Sports Sciences” and “J Sports Sci”, were unified as “J Sports Sci”.
- We merged synonymous keywords to avoid redundancy. For example, “Computer analysis”, “computered analysis”, and “computerized analysis” were consolidated under “computer analysis”.
- Supplementing missing information by manually filling in incomplete fields, including author names, publication years, and keywords. Citation data and reference details for publications imported from PubMed were supplemented with information from original documents and Google Scholar.

## 3. Results and Analysis

### 3.1. Research Progress

#### 3.1.1. Quantitative Description

After automatic processing and manual screening, this study included 206 publications (201 research articles and 5 review articles). These publications show an average annual output of 3.89 articles and reflect contributions from 36 countries, 206 institutions, and 470 authors. The research was published across 115 journals, generating 683 unique keywords. Additionally, these studies cited 3452 references authored by 2543 individuals, sourced from 1458 journals.

#### 3.1.2. The Overall Trend of Publications and Distribution of Disciplinary Fields

**Figure 2:**
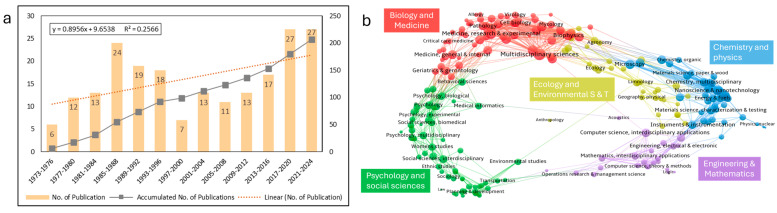
The overall trend of publications (**a**) and disciplinary distribution (**b**).

The number of publications has been used as a form of recognition in scientific research [17], as well as an essential indicator of productivity and performance of the field [18]. Research on squash can be traced back to 1973. Given the extended period span, this study used four-year intervals to depict the trend in publication volume (Figure 2a). The number of publications remains relatively low but shows a gradual upward trend. A notable increase after a decline between 1997 and 2016, indicates a growing research activity and attention. However, regression analysis suggests a weak linear relationship between publication output growth and time (R^2^ = 0.2566), implying that additional factors may influence this trend.

The disciplinary distribution of squash research indicates that 206 studies were spread across 49 fields (Figure 2b). Biology, medicine, and psychology and social science account for a relatively high proportion. The top contributing field is sports science (73 publications, 35.4%), followed by medicine (64 publications, 30.9%) and health professions (37 publications, 17.9%).

#### 3.1.3. Analysis of Main Research Journals

The number of publications indicates the volume of research output, while citations are considered as objective markers of research quality and significance, with higher citation counts suggesting greater impact [19]. Table 1 lists the top ten journals in terms of publication volume. These 11 journals collectively published 64 articles, accounting for 30.9% of the total publications, making them core journals in squash research. The *Journal of Sports Sciences* and the *British Journal of Sports Medicine* stand out with 14 and 12 publications, respectively. These two journals are ranked in Q2 and Q1 in the JCR (Journal Citation Reports) categories. They have average citation counts of 56 and 20 per article, reflecting their high quality and influence in the field. The remaining eight journals also show significant contributions. While their citation rates are slightly lower than the top two, they are essential in specialization and field coverage. For instance, journals like the *Journal of Strength and Conditioning Research* and the *International Journal of Sports Physiology and Performance* provide researchers with diverse platforms for publication across various subfields. This distribution implies that researchers prioritize journals’ academic reputation and field relevance when selecting publication venues.

In brief, squash research has gradually increased over the past five decades but remains limited in volume and global visibility. The field demonstrates interdisciplinary integration, primarily involving sports science, medicine, and health professions. Most publications appear in journals related to sports science and sports medicine.

### 3.2. Research Cooperation of the Publications

**Figure 3:**
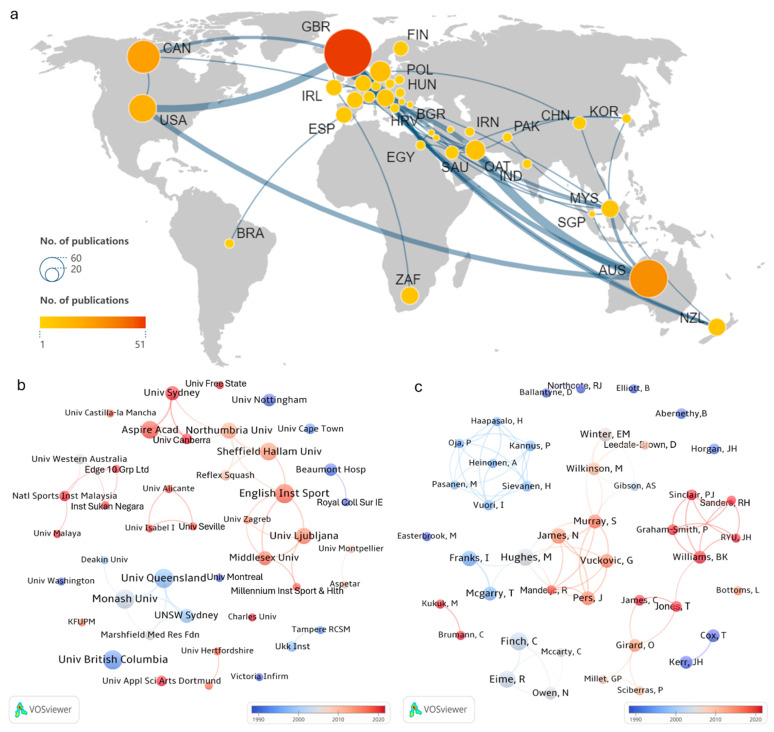
The collaboration networks among countries (**a**), institutions (**b**), and authors (**c**).

The co-occurrence network highlights the contributions and collaborative roles of countries, institutions, and researchers in scientific research, reflecting academic partnership’s global distribution and intensity [13]. Figure 3 visualizes collaboration networks among countries (3a), institutions (3b), and authors (3c) in squash research. Node size corresponds to publication volume, while link thickness represents the frequency of collaboration. Larger nodes and thicker links highlight dominant contributors and close collaboration patterns. This visualization supports identifying of key knowledge producers and the collaborative dynamics within squash research [13].

#### 3.2.1. Country Collaboration Network

Squash research has garnered global attention, yet the distribution of international collaborations exhibits a clear core-periphery structure. The United Kingdom (largest node) and Australia (second-largest node) occupy the central positions in the collaboration network (Figure 3a), evidenced by their thickest links to 28 and 14 partner countries, respectively (Table 2). Canada (third-largest node) and the United States (USA) rank among the top contributors but exhibit narrower collaboration networks (indicated by thinner links), primarily partnering with the UK and Australia. European nations (Germany, Netherlands, France) form a highly interconnected network. Smaller nodes represent emerging contributors like Qatar and Malaysia but maintain strategic ties to core nations (e.g., UK-Australia network), signaling their integration into global knowledge flows.

#### 3.2.2. Institutional Collaboration Network

Figure 3b visualizes institutional collaborations (co-authorship frequency ≥ 2) and overlay mapping, illustrating the research activity’s structure and temporal evolution. The color gradient from blue to red represents the average year of publication, with blue indicating earlier years and red denoting more recent years. Early research (blue nodes, pre-2005) featured independent research patterns like the University of British Columbia and the University of Nottingham, which did not engage in external collaborations. Major research institutions during this period included the University of Queensland, Monash University, and the two mentioned above. Notably, the University of Queensland focused primarily on domestic partnerships. Since 2010 (red nodes), institutional collaborations expanded significantly: the English Institute of Sport, Sheffield Hallam University, and Northumbria University emerged as central nodes, characterized by larger node sizes and stronger connections, reflecting higher output and broader collaboration. Additionally, the entry of newer institutions, such as the University of Ljubljana in Europe, the Aspire Academy in the Middle East, and the Malaysian National Sports Institute in Southeast Asia, has contributed to greater geographic diversity and knowledge exchange.

#### 3.2.3. Author Collaboration Network

Figure 3c presents the overlay mapping of author collaboration networks (co-authorship frequency ≥ 3), highlighting key research teams and their collaborative dynamics. The largest and longest-standing collaboration network is centered around Mike Hughes (largest node) and Stafford Murray (second-largest node) and spans from 1995 (blue) to 2020 (red), reflecting sustained productivity. Among the 12 scholars in this network, eight rank among the top 10 prolific authors (see Section 3.2.6), underscoring their sustained leadership in squash-related research. Another prominent network, led by Williams and Jones (medium-sized nodes) emerging later (red nodes), demonstrates focused collaboration on specific research themes. In contrast, transient collaborations (smaller networks, small or isolated nodes, thin links) or isolated nodes exhibit distinct phase-specific characteristics with limited collaboration intensity and duration, which have not yet evolved into stable research groups.

#### 3.2.4. Top 10 Countries by Publication Volume

Academic power and influence in a given field can be assessed based on the quantity of publications and citations [13]. The UK ranks first with 51 publications (Table 2), significantly surpassing Australia (31), Canada (21), and the USA (16). This underscores the UK’s academic leadership in this field. Additionally, the average citation counts per publication for Australia (30.81), Canada (32.76), and the USA (32.81) rank among the top three. These figures highlight that the academic influence of these countries is reflected not only in their publication volumes but also in the quality of their research.

#### 3.2.5. Top 10 Institutions by Publication Volume

The top 10 institutions in squash research are all higher education institutions, primarily in countries with active international collaborations (Table 3). The University of Queensland (eight publications, average citations of 95.50) and the University of British Columbia (eight publications, average citations of 67) hold leading positions. Other institutions, such as Monash University (eight publications, average citations of 17.5) and the English Institute of Sport (eight publications, average citations of 7.5), also demonstrate strong research output. However, their relatively lower average citation counts suggest their research impact may be limited to specific subfields.

#### 3.2.6. Top 10 Authors by Publication Volume

Table 4 reveals that many prolific authors have international research backgrounds. Mike Hughes (eight publications, average citations of 51), Ian Franks (seven publications, average citations of 75.14), and Tim McGarry (six publications, average citations of 84.17) are among the most influential scholars. According to Price’s Law [20], the threshold for core authorship is set at three publications ($N=0.749\cdot \sqrt{9}=2.25$). This study identified 47 core authors (Figure 3c), accounting for 10.06% of the total, far below the expected 50% threshold for a mature research field. Additionally, authors with only one publication account for 79.66% (372 individuals), significantly exceeding the 60% benchmark suggested by Lotka’s law. These findings indicate that the core research team in squash studies is not yet well-established, and research output heavily relies on few researchers.

Global research collaborations exhibit a hierarchical structure, with authors and institutions from the UK, Australia, and Canada occupying leading positions. While established partnerships contribute to research productivity, future development requires broader international participation and stronger interdisciplinary exchange.

### 3.3. Research Findings

#### 3.3.1. Co-Citation Cluster Analysis

**Figure 4:**
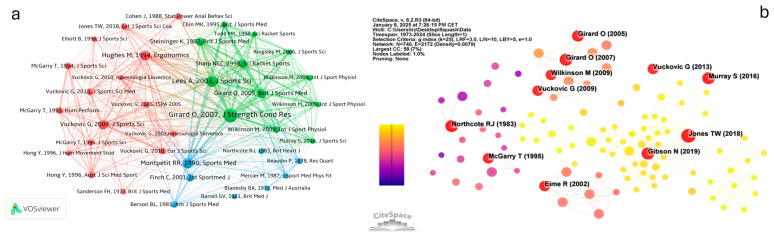
Co-citation literature mapping. (**a**) Clustering mapping of references. (**b**) Burst detection of co-citations.

Highly co-cited references are often considered foundational academic resources in the research field [20] and can be utilized to investigate knowledge structure and research boundaries [21]. This study constructed a co-citation network using references with citation frequencies ≥ 7 (Figure 4a). The size of the nodes represents the citation frequency, the color reflects the year in which co-citations occurred, and the links denote co-citation relationships. The width of the edges indicates the strength of these relationships: the wider the edge, the stronger the connection. The co-citation analysis reveals that highly co-cited literature in squash research is primarily concentrated on the following three areas:

1. Cluster 1 (red, 16 studies): Performance Analysis. This cluster focuses on the measurement or analysis of the player’s performance from a data-driven perspective, including tactical and technical analysis, mathematical and computer modeling, and dynamic pattern analysis. For example, Hughes and Franks [22] were the first to reveal dynamic pattern characteristics in squash matches. In subsequent studies they later developed the theoretical framework of “dynamic is a self-organized system” [23]. These works provide the theoretical foundation for tactical and technical analysis and serve as key references for performance analysis research.
2. Cluster 2 (green, 12 studies): Physiology and Physical Fitness. This cluster concentrates on squash players’ physiological and fitness demands, including monitoring physiological parameters and methods for fitness training and testing. For example, studies by Girard et al. [24,25] introduced squash-specific fitness testing methods and revealed their energy demand characteristics. These findings provide a scientific basis for optimizing training programs and advancing fitness science.
3. Cluster 3 (blue, eight studies): Clinical Research and Safety. This cluster primarily covers studies related to sports injuries and injury prevention, including topics such as eye injuries and cardiovascular risks. For example, research by Northcote et al. [26] and Finch and Vear [27] identified common squash-related injuries and proposed preventive measures. These works have laid the theoretical foundation for the epidemiologic study of squash injuries.

Cluster 1 contains the most significant number of references, indicating the extensive focus on performance analysis in squash research. The references in Cluster 2 exhibit the closest co-citation relationships, underscoring the importance of physiological and physical fitness studies. These clusters reflect squash’s three main research areas and multidisciplinary nature.

#### 3.3.2. Burst Co-Citation Analysis

By identifying burst literature, it is possible to analyze highly influential research and its impact on subsequent studies [13]. Figure 4b presents the burst detection map of squash research, where nodes with burst characteristics are filled in red. Table 5 lists the top ten burst literature items, with red segments indicating the burst periods. The burst time spans from 1983 to 2024. These 10 co-citation burst literature items belong to the three areas depicted in Figure 4a, showcasing distinct thematic and temporal characteristics.

Research before 2002 primarily focused on health risks during or after squash exercise [26] and protective measures, particularly eye protection [29]. Between 2003 and 2013, research expanded from foundational physiological studies to energy demands, fitness testing and assessment [24,25], and technical and tactical analysis [30,31]. These works highlighted the interplay between fitness demands and tactical decision-making, providing insights into the strategies employed by squash players. Since 2014, research has gradually shifted toward optimizing training methods and identifying key factors for performance improvement. Studies by Gibson et al. [33], Jones et al. [4], and Murray et al. [32] have examined performance enhancement from the perspective of physical requirements and preparation. These studies provide practical guidance for elite athlete training, advancing squash professionalization and scientific development.

#### 3.3.3. Top 10 Co-Cited References

Table 6 lists the 10 most frequently co-cited references. Among them, five pertain to physiology and physical fitness (Cluster 2), three focus on performance analysis (Cluster 1), and two are categorized under clinical research and safety (Cluster 3). Four of these references are also identified as burst references, highlighting their widespread academic influence. From a chronological perspective, these ten highly cited references span 26 years and were published across 10 years, with intervals of 2–4 years. This suggests that knowledge production in this field is steady but progressing slowly. Seven of these references were authored by researchers ranked among the top 10 in terms of publication volume, further emphasizing the pivotal role of a few key scholars in advancing the field. The most recently highly cited reference was published in 2013, possibly because more recent research from the past decade has not yet accumulated sufficient citations. This may also indicate a recent lack of “transformative” or “breakthrough” studies.

Co-citation analysis identifies three main thematic clusters: performance analysis, physiology and fitness, and clinical research and safety. The co-citation frequency in the first two categories is significantly higher than in the latter. Citation burst analysis reveals evolving research priorities, shifting from early focus on health risks to more recent emphasis on performance enhancement. The lack of recent highly cited studies points to slow progress and a shortage of breakthrough work, suggesting a need for fresh directions and innovation in the field.

### 3.4. Research Thematic Evolution

#### 3.4.1. Research Hotspots

**Figure 5:**
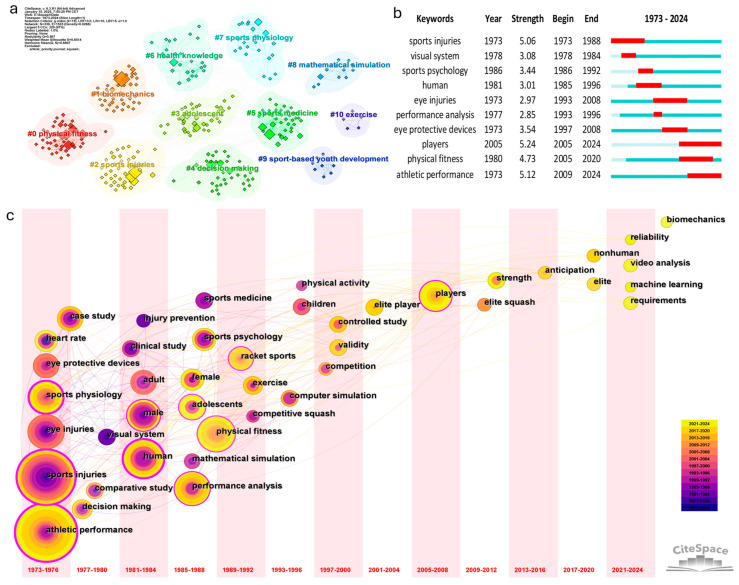
Keywords clustering (**a**), the top 10 keywords with the most vigorous citation bursts (**b**), and the time-zone evolution mapping (**c**).

Keywords reflect the themes and research focus of the study. Analyzing the frequency and distribution of keywords can help identify trending topics and their dynamic changes [13]. Figure 5a presents the clustering analysis of all the keywords in the 206 studies. It illustrates the diversity of research topics and the relationships between clusters. The clustering network generated using CiteSpace (Modularity Q = 0.567, Silhouette S = 0.8514) indicates a well-structured network with high internal consistency and relative independence among clusters. These eleven themes represent the primary research topics in squash. Table 7 lists the core keywords in each cluster. Based on the intrinsic connections and interdisciplinary nature of the research content, they can be further consolidated into five research areas:

Athletic Performance (Clusters 0, 1, and 7). Research on athletic performance constitutes the core area of squash studies. Cluster 0 focuses on players’ physical training. Numerous scholars have quantified the physical demands of play through various fitness and physiological “tests” assessing parameters such as “physical endurance” and “training load”. These studies have validated the effectiveness of existing fitness tests [38,39] and identified the most efficient training methods [33]. Although “biomechanics”, the keyword of Cluster 1, emerged relatively late, research on “short-duration explosive effort” dates back to 1985 [40]. This type of study improves movement efficiency through technical analysis of specific movements, such as forehand drive, “fencing lunge” [41,42,43], or the “coordination” during the “phase transition”. Physiological studies in Cluster 7 examine factors such as “body fluids” [44], “intensity”, “blood pressure”, and cardiovascular adaptation [25,45]. These studies explore the mechanisms of maintaining physiological homeostasis during high-intensity competition [46], providing physiological insights into endurance enhancement and movement efficiency. These three themes aim to optimize player’s performance regulation systems, enhancing their physical adaptability, movement coordination, and physiological reserves. By employing scientific training methods, researchers seek to improve players’ sustained performance capacity and movement efficiency during matches, offering a physiological and training basis for squash.

Sports medicine and Safety (Clusters 2 and 5). Cluster 2 addresses squash-specific injuries [47,48,49,50], with “eye injuries” being the most frequently studied topic, which could be traced back to the origins of squash research [51]. Consequently, “epidemiological studies” on squash-related injuries [52,53] and research on “eye protective devices” [54,55] remained central themes over the following three decades. Cluster 5 (sports medicine) covers studies involving “human experiments” [56], “case studies” [57,58], and physiological monitoring [59], highlighting the medical and rehabilitation aspects of squash research. These two clusters underscore the significance of injury risk assessment and prevention in squash.

Cognitive and performance analysis (Clusters 4 and 8). Cluster 4 (decision-making) primarily encompasses two types of research. The first focuses on “sports psychology”, where psychologists examine the cognitive aspects of gameplay, including decision-making processes [60], “performance analysis” [28], and anticipatory skills [61] in squash. This research bridges cognitive science and tactical execution, emphasizing anticipation skills and psychological resilience under competitive pressure [62]. The second research type involves performance analysis through “mathematical simulation” as well as computational simulation (Cluster 8). Analysts use modeling techniques to study decision-making differences in tactical scenarios [31], match analysis [22], and predict winning probabilities [63] and match outcomes [64]. Artificial intelligence (AI) technologies, such as “machine learning” and “heatmaps”, have also been applied to player tracking [65,66] and refereeing [67].

Youth training and development (Clusters 3 and 9). Studies in cluster 3 suggest that early participation in squash contributes to bone mineralization in childhood and adolescence [68,69], which promotes physical growth [70]. Cluster 9 (sport-based youth development) explores the role of squash in youth development. It highlights that the program provides high-quality squash activities during “out-of-school time”, enhancing academic performance and promoting personal and social responsibility. These benefits can also be transferred to daily school learning [71].

Health promotion (Cluster 6 & 10). Cluster 6 (health knowledge) relates to “attitudes toward health”, i.e., injury prevention [72], sports nutrition knowledge of elite squash players such as healthy diets [73], nutritional supplement consumption [74], and nutritional interventions [58], linking squash participation to broader health benefits. Cluster 10 (exercise) focuses on health promotion, disease prevention, and growth-related benefits [68,75].

**Table 7:** Core keywords in each cluster.

Cluster No.	Theme	Core Keywords
0	physical fitness	test, physical endurance, training load
1	biomechanics	fencing lunge, coordination, phase transition
2	sports injuries	eye injuries, eye protective devices, injury prevention
3	adolescent	children, skill, bone density
4	decision making	performance analysis, sports psychology, anticipation
5	sports medicine	human, human experiment, case study
6	health knowledge	attitude to health, diet, fatty acid
7	sports physiology	body fluids, intensity, blood pressure
8	mathematical simulation	machine learning, gaussian distribution, heatmap
9	sport-based youth development	physical education, out of school time, goals
10	exercise	health promotion, disease prevention, growth

#### 3.4.2. Evolution Analysis of Research Hotspots

The burst keywords (Figure 5b) and time-zone evolution mapping (Figure 5c) reveal the dynamic evolution of research hotspots in squash. The evolution of research themes can be divided into three stages:

Early Stage (1973–1987): Sports injuries and physiological monitoring. Research hotspots focused on “sports injuries”, particularly the epidemiology of “eye injuries” [51,76] and cases of sudden death [57] investigated through “clinical studies” and “case studies”. The research subjects mainly were “male” and “adults”. Keywords such as “sports injuries” and “visual system” exhibited the highest burst frequencies during this period. Additionally, researchers began to monitor physiological and medical indicators in players [26,59], such as “heart rate”, blood pressure, and blood lactate levels, laying a scientific foundation for sports safety research.

Transition Stage (1988–2004): Physical fitness, psychological and performance analysis. The research focused on “physical fitness” and “sports psychology”, making these this period’s dominant themes. Studies expanded to include the performance of “female” [70] and “adolescent” players. The benefits of squash exercise for children’s physical development were also explored [69]. Additionally, “performance analysis” in “competitive squash” also emerged during this stage, with decision analysis [63], “mathematical simulation” [28], and “computer simulation” [77] serving as primary analytical methods. Studies on sports injuries gradually shifted toward injury prevention, particularly emphasizing eye injuries and the development of eye protective devices [29].

Modern Stage (2005–Present): Performance optimization and artificial intelligence integration. Since 2005, research has increasingly emphasized performance optimization and precision-based training. Keywords such as “player”, “physical fitness”, and “athletic performance” have exhibited persistent citation bursts during this period. Studies have delved deeper into the “strength”, “anticipation”, “biomechanics”, and performance “requirements” of “elite squash” players. In recent years, the application of AI technology, particularly “machine learning”, has significantly advanced player’s real-time position tracking and motion analysis [65,66]. Breakthroughs in “video analysis” have provided valuable technological support for scientific training methodologies.

The keyword-based thematic evolution analysis shows that squash research has transitioned from early concerns with sports injuries and physiological monitoring to current interests in athletic performance enhancement and technological innovation, including AI. Underexplored topics such as youth development, sports psychology, and nutrition present essential directions for future exploration.

### 3.5. Research Maturity

**Figure 6:**
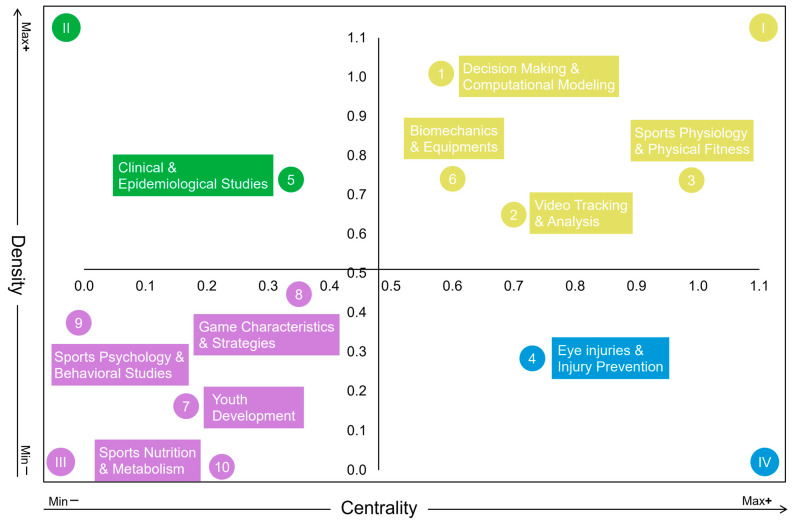
Strategic diagram of theme clusters. The horizonal axis (centrality) indicates the position of a theme within the field. Higher centrality values suggest closer connections to other themes and a more central research position. The vertical axis (density) reflects the internal development of a theme. Higher density values denote greater research maturity. The themes were drawn based on 96 high-frequency keywords (frequency ≥ 3), grouped into 10 clusters (labeled 1–10) via co-occurrence analysis. A lower cluster number corresponds to a larger cluster size. The coordinates of the origin were normalized to (0.51, 0.48).

Figure 6 presents the strategic diagram generated through keywords co-occurrence analysis. By visualizing themes along the dimensions of centrality and density, this diagram helps reveal which topics are well-developed and integral to the field and which remain peripheral or underdeveloped. The thematic positioning offers insight into the intellectual structure and potential research priorities within squash studies [78].

Quadrant I (high density–high centrality): This quadrant includes cluster 1 (decision making and computational modeling), Cluster 2 (video tracking and analysis), cluster 3 (sports physiology and physical fitness), and cluster 6 (biomechanics and equipment). These themes exhibit strong internal research connections while maintaining close associations with others, making them the core and most mature areas in squash research.

Quadrant II (high density–low centrality): Cluster 5 (clinical and epidemiological studies) is the sole theme in this quadrant. While this theme is relatively mature and has strong internal connections, it has limited associations with other research themes.

Quadrant III (low density–low centrality): This quadrant comprises cluster 7 (youth development), cluster 8 (technical and tactical analysis), cluster 9 (sports psychology and behavioral studies), and cluster 10 (sports nutrition and metabolism). These themes are underdeveloped with limited internal and external connections, indicating that they have not yet developed into sustained research hotspots and are considered emerging or marginal areas.

Quadrant IV (low density–high centrality): Cluster 4 (eye injuries and injury prevention) falls into this quadrant. It maintains strong connections with other research themes but is still underdeveloped in internal research, indicating that it is in the immature development stage.

To sum up, core themes in squash research include athletic performance, physical fitness, and data-driven modeling and analysis. Topics related to sports injuries and injury prevention remain highly interconnected with other themes and hold significant potential for future development. Emerging areas, such as technical and tactical analysis, youth player development, psychological studies, and sports nutrition, have received insufficient academic attention and warrant deeper investigation.

## 4. Discussion

### 4.1. Research Progress

#### 4.1.1. Publication Volume

Squash has a 53-year research history, but its overall publication volume remains relatively low compared to collective sports [79] or other racket sports [9,80]. A significant increase was observed during 1985–1988 and 2013–2016 (Figure 2a), which is likely linked to the sport’s professionalization and growing global influence. For instance, in 1986, the IOC granted official recognition to squash [81], leading to the emergence of research across various fields, including sports medicine, performance analysis, sports psychology, physiology, and physical fitness. The 2014 Glasgow Commonwealth Games marked a breakthrough in media coverage and match broadcasting, increasing the sport’s accessibility and attractiveness to a broader audience, including researchers [82]. These external factors may have contributed to the increase in research output.

#### 4.1.2. Disciplinary Distribution

Squash research exhibits a distinctly interdisciplinary nature, primarily focusing on sports science and physical education while also integrating health sciences, psychology, training optimization, and sports injury prevention (Figure 2b). Sports science and physical education dominate the research landscape, likely due to its technical complexity and high physical demands. Health science research primarily addresses common squash-related injuries and prevention strategies, including studies on eye protection devices and cardiovascular health monitoring [26,27]. AI has emerged as a key research tool in recent years, particularly in technical and tactical analysis and training optimization (Figure 4a; [24,31]. These interdisciplinary integrations not only highlight the complexity of squash research but also provide opportunities for future academic expansion.

Scientific research on squash remains in its early exploratory stage and has yet to receive sufficient attention from the academic community. As squash was not included in the Olympic Games until 2028, this may have constrained the scale and depth of research in this field.

### 4.2. Research Collaborations

#### 4.2.1. National Collaboration

Squash research exhibits significant national clustering patterns, predominantly led by the UK, Australia, and Canada. These countries lead in publication volume and demonstrate substantial academic influence through high citation counts (Table 2). Their prominence can be attributed to their well-established sports science research environments, the widespread popularity of squash [2], and robust academic networks (Figure 3). For instance, the UK is a central node across 28 international collaborations, while Australia and Canada demonstrate high research quality and citation impact. Emerging countries and institutions are gaining recognition in squash research. For example, Qatar’s Aspire Academy and Malaysia’s National Sports Institute have established strong connections with multiple international partners, indicating considerable developmental potential. These emerging forces enhance the connectivity of global collaboration networks, fostering international academic exchange and the development of squash research.

#### 4.2.2. Institutional Collaboration

Regional collaboration among institutions is a notable feature of squash research. The English Institute of Sport, Sheffield Hallam University, and Northumbria University have formed stable research clusters in the UK. Similarly, Australian institutions, including The University of Queensland, Monash University, and UNSW Sydney, along with Spanish universities, such as the University of Alicante, Universidad Isabel I, and the University of Seville, have established consistent research networks. Top-tier institutions like The University of Queensland and The University of British Columbia play pivotal roles in their regions. These regional collaborations enhance research quality and facilitate knowledge dissemination and sharing (Figure 3b).

#### 4.2.3. Author Collaboration

The author’s collaboration network clearly groups researchers. Leading scholars like Mike Hughes and Stafford Murray are central figures linking different research teams (Figure 3c). These core authors enhance network connectivity and drive the development of emerging research topics through cross-team collaboration. However, the isolated nodes and limited inter-cluster connections suggest room for improvement in global and institutional cooperation and interdisciplinary integration.

Furthermore, the institutional mobility of prolific authors is another factor contributing to international and institutional cooperation. Among the top 10 authors by publication volume, only Ian Franks, Janez Pers, and Goran Vuckovic consistently maintain affiliations with a single institution. In contrast, the remaining authors have two or more corresponding affiliations. Williams, Benjamin K. listed Aspire Academy and The University of Sydney as corresponding affiliations in all five publications. This institutional mobility has facilitated knowledge and resource exchange across regions, further enhancing collaboration and the global integration of squash research.

Higher socio-economic status is strongly associated with increased sports participation and physical activities [83]. Developed nations like the UK, Australia, and Canada have made substantial government investments in sports infrastructure. Within these societies, sport participation, such as squash, has become normative forms of behavior that self-regulate an individual’s leisure preferences [84]. These factors are likely to contribute to higher public interest in squash in these countries, consequently attracting greater attention from researchers. In contrast, in economically developing regions (e.g., China, Egypt, South Africa), national sports policies prioritize Olympic-medal-winning disciplines. Non-Olympic sports like squash receive less support, media coverage, and financial investment [85,86]. This Olympic-centric approach severely constrains scientific research progress in squash, creating a disparity between competitive success and research output in regions like Egypt.

Considering the above, the collaboration networks of countries, institutions, and authors in squash research show a highly centralized characteristic. A few highly productive countries (such as the UK and Australia), influential institutions (such as The University of Queensland and The University of British Columbia), and core authors (such as Mike Hughes and Stafford Murray) constitute the core driving force in the field. This centralized research network fosters scientific progress through efficient knowledge dissemination and innovative systems. However, certain limitations remain. Such concentration may restrict the development of a global perspective and limit the diversity of research topics. Moreover, overreliance on a small group of prolific authors could reduce the academic engagement of researchers from less-represented regions, potentially limiting the breadth and innovation of research. Future studies should focus on enhancing participation from emerging countries, expanding international collaboration and promoting interdisciplinary integration, particularly in adopting new technologies (e.g., AI and big data analytics) to improve globalization and diversity of research. Additionally, encouraging the involvement of more fresh scholars will be essential for fostering sustainable and innovative development in squash-related science.

### 4.3. Research Findings

The analysis of co-citation burst co-citation and top 0 co-cited references highlight milestone contributions within the field. These references have profoundly influenced theoretical deepening and practical applications, driving the evolution of research and offering valuable insights into areas such as performance optimization, injury prevention, and training methodologies.

Technological advancements in research. These references have facilitated the reform and innovation of research technologies. Vuckovic et al. [30,31] developed the SAGIT/Squash automated player-tracking system to quantify players’ movements during the match. This system marked a paradigm shift from traditional hand notational analysis to computerized data collection and analysis, significantly improving research accuracy and efficiency. Building on this foundation, Murray et al. [32] employed the Tracker System, an advanced SAGIT version, to quantitatively analyze players’ physical demands and shot characteristics following the 2008 rule changes. Their work validated the feasibility of technological applications in squash research and provided empirical evidence supporting rule reforms. These cases highlight the critical role of modern research technologies in optimizing training, refining match strategies, and influencing policy decisions.

Theoretical developments and expansion. Some key references have contributed to establishing and expanding theoretical frameworks in squash research. McGarry and Franks [28] introduced the Markov model, elevating squash match behavioral analysis to a predictive level. Their “consistent and inconsistent behavioral analysis” theory enriched the theoretical foundations of squash match analysis and provided valuable insights for other sports (e.g., football, basketball, tennis, gymnastics, and swimming). Jones et al. [4] synthesized research findings on squash performance, identified critical performance evaluation metrics, and proposed future research directions, offering the academic community a more comprehensive theoretical perspective.

Practical applications in training and health management. These references carry notable practical significance. Girard et al. [24,25] investigated the physiological demands and energy requirements of squash through specialized fitness tests, while Gibson et al. [33] systematically examined elite players’ physical preparation from the perspectives of monitoring, assessment, and training methodologies. Their findings provide a crucial reference for evidence-based training practices. Furthermore, Northcote et al. [26] highlighted the cardiovascular risks of squash, issuing an essential warning for health management among high-risk populations. Eime et al. [29] conducted a large-scale survey on goggles in injury prevention, identifying key influencing factors. These studies demonstrate that squash research enhances athletic performance and plays a proactive role in health protection.

These milestone references have advanced both theoretical and practical dimensions of squash research, propelling innovation by integrating new technologies and methodologies. Nevertheless, current research predominantly centers on professional players and specialized topics, with insufficient attention to general participants, long-term health implications, and interdisciplinary integration.

### 4.4. Research Thematic Evolution

#### 4.4.1. Research Hotspots

Keyword clustering analysis indicates that squash research has formed a stable core theme centered on athletic performance enhancement. Several subfields have been established to support this objective, including physical fitness, biomechanics, sports physiology, performance analysis, sports psychology, and sports medicine. These subfields collectively provide theoretical foundations and practical applications for the scientific development of squash. Beyond elite performance, other research topics have gained temporary academic attention, including the role of squash in promoting public health [87], its influence on youth development and growth [69] and its feasibility as an extracurricular activity [71]. These studies consistently highlight the social benefits of squash, reinforcing its value beyond professional sports. Nevertheless, its broader social impact remains underexplored and underdeveloped.

#### 4.4.2. Research Evolution

Keyword burst analysis and evolution mapping reveal a distinct shift in research focus from health and safety to performance enhancement. Early studies primarily addressed sports injuries [52] and cardiovascular health [26]. These investigations improved player safety [88] and advancements in health monitoring technologies [59]. Subsequent research progressively examined the impact of physical fitness [36] and psychological factors [89] on performance. The introduction of innovative methodologies such as computer simulation [77] and dynamic pattern analysis [22] stimulated the development and iterative improvement of performance analysis tools and software [31,90,91]. In recent years, AI-based algorithms, including neural networks (e.g., multilayer perceptron, convolutional neural networks) and evolutionary methods (e.g., genetic algorithms), have been applied to tasks such as player tracking [65] and motion analysis [66]. Emerging research areas, such as AI-assisted refereeing, have begun to develop [67]. However, the application of AI is still limited, with only five studies involving AI, three of which were conducted by Brumann’s group.

This evolution reflects the dynamic growth of squash research, which has advanced from foundational safety concerns to state-of-the-art performance enhancement strategies, driven by technological innovation and interdisciplinary integration. This transition reflects the progressive deepening of research themes and the growing integration between theory and practice.

### 4.5. Research Maturity

Themes directly related to improving athletic performance, such as decision making and computational modeling, video tracking and analysis, and sports physiology and physical fitness, are now the core of squash research. In contrast, research on clinical and epidemiological studies exhibits high density and maturity but remains a relatively independent academic ecosystem. This is likely due to its focus on specific physiological mechanisms, clinical trials, and medical interventions, which inherently differ from other topics such as technical and tactical analysis. The themes of game characteristics and strategies, youth development, sports psychology, and sports nutrition have very limited publication volumes, with most studies dating back over a decade. This suggests that these areas have not yet established stable research communities or continuity, highlighting room for future exploration. These gaps may provide critical opportunities for breakthrough research. Eye injuries and prevention were research hotspots in the early years of squash studies. There are 31 papers published on squash between 1973 and 1984 (Figure 2a), 15 of which focused on sports-related injuries, with 10 explicitly addressing visual system damage or protection. With the increasing calling for the use of eye protective devices [54,55,88] and the growing professionalization of the sport, players’ awareness of protection has gradually improved. Consequently, the focus on squash-related injuries has decreased, and the publication volume on this topic has noticeably declined.

Emerging topics in squash research remain weakly developed, which can be attributed to several reasons. First and foremost, there is a lack of dedicated research funding. Governments often prioritize investments in sports that yield immediate international prestige, such as elite sports or Olympic events, rather than non-Olympic events like squash [86,92]. Consequently, studies involving psychological experiments, nutritional reagent interventions, or AI-enhanced match analyses, which require substantial resources for specialized equipment and technology, are less likely to receive support. Second, despite squash’s popularity among recreational players in Europe, the USA, and Australia, its professionalization remains limited. Low broadcast coverage and media exposure of tournaments [93] restrict researchers’ access to the match data needed for analysis. Third, squash carries a relatively high risk of injury, and teachers tend to favor safer activities in physical education classes where one teacher faces multiple students [94]. This safety concern may limit squash’s inclusion in youth program and thus constrain its research pipeline.

In summary, squash research maturity demonstrates a clear hierarchical structure: core research themes focus on improving the performance of professional players. At the same time, other areas have not received sufficient attention. Future research should maintain this core focus while expanding into peripheral but essential topics, such as exploring the developmental mechanisms of youth players and the impact of psychology and nutrition on squash performance. This will broaden the scope of squash research and provide more comprehensive support for the overall optimization of athletic performance.

### 4.6. Limitations

Scope of data selection. The data for this study were primarily sourced from the Web of Science Core Collection, Scopus, and PubMed, focusing on articles and review articles published in English. This selection excluded specific research outputs, such as conference papers, non-English publications, and monographs. Consequently, emerging or preliminary research may have been overlooked, potentially biasing our analyses of publication trends, thematic evolution, and identifying of nascent topics. Thus, the findings may not fully capture the breadth of squash research globally.

Literature inclusion criteria. The study prioritized highly cited papers during the analysis as they are generally considered to hold significant academic value. However, low-citation or uncited papers may also contain important findings that were not accounted for. Additionally, only keywords with a frequency of ≥3 were included when conducting research hotspot identification, keyword clustering, and strategic mapping analysis. This may result in the omission of low frequency but potentially pioneering or emerging keywords, which could limit the ability to capture emerging themes fully.

### 4.7. Implications

Over the past 52 years, the development of squash research has been slow, yet significant progress and breakthroughs have been made in various areas. Research subjects have expanded from adult professional players to university students [87] and non-athletes [95]. Research methods have evolved from traditional mathematical and theoretical simulations [96] to approaches emphasizing technical and tactical elements [97]. Theoretical frameworks have advanced from the conventional Markov model [28] to complex algorithms integrated with AI technologies [66]. Data collection and evaluation methods have transitioned from manual measurement and collection [98] to automated tracking and analysis relying on high-tech equipment [90,99]. Despite these strides, squash research remains nascent compared to popular Olympic sports, and there is still vast potential for future development.

1. Expanding the breadth and depth of research topics. Future research should extend beyond the current focus on enhancing competitive squash performance and explore more under-researched areas. Potential topics include professionalization and promotion strategies for squash events; training and career pathways for referees; the popularization and promotion in recreational sports and school education; and research on pedagogy for different groups. Addressing these research gaps would broaden the scope of squash studies and provide theoretical support for its global popularization.
2. Diversifying research subjects. Future studies should broaden the focus from primarily adult professional players to include youth, recreational enthusiasts, and general participants. Research should address the needs of players at different levels by developing tailored training recommendations, performance evaluation methods, and participation strategies. As a high-intensity aerobic sport, squash may significantly benefit cardiovascular health, weight management, and overall physical well-being [87]. Investigating these aspects could provide scientific evidence to support the promotion of squash as a health-enhancing activity, further enhancing its social value.
3. Establishing comprehensive youth development mechanism. Future initiatives should focus on creating structured pathways for youth participation by integrating squash into school physical education curriculum and implementing standardized talent identification protocols. National federations should develop multi-tiered development systems with clear progression routes from grassroots to elite competition. Age-appropriate coaching methodologies and long-term talent development models should support these systems. Furthermore, public-private partnerships could be key in expanding facility access, especially in underserved communities, through innovative court designs requiring less space and lower construction costs.
4. Promoting the deep integration of science and technology in squash research. Future research should strengthen interdisciplinary collaboration by incorporating theories and methods from various fields, particularly leveraging advancements in AI to drive innovation in squash studies. For example, real-time training monitoring systems and intelligent decision analysis systems could be developed by merging sports physiology with wearable equipment and machine learning algorithms; virtual reality–based mental training platform may also enhance tactical awareness and psychological resilience, not only for professional players but also for beginners and recreational participants, by making skill acquisition more engaging and accessible. In this way, scientific innovation can serve both the high-performance domain and broader recreational and educational contexts, supporting evidence-based training, skill development, and sport promotion through multidisciplinary synergies.

## 5. Conclusions

This study conducted a quantitative and qualitative analysis of 206 publications on squash scientific research through a bibliometric approach, focusing on four dimensions: research progress, research collaboration, research findings, and thematic evolution. The study highlights the strengths and gaps in current squash research, providing valuable insights into the field’s evolution and future potential. The following four main conclusions are drawn:

1. Scientific research on squash demonstrates limited productivity with merely 3.89 papers published annually, substantially lower than the comparative racket sports research output. Publications predominantly cluster in sports science, medicine, and health professions. The *Journal of Sports Sciences* and *British Journal of Sports Medicine* emerge as leading published journals, along with other international journals from the UK and the US specializing in sports medicine, physical fitness, and performance analysis.
2. Western developed countries primarily drive research on squash. The UK is first in both publication volume and international collaboration, followed by Australia. Australia, Canada, and the US hold the highest average citation rates, indicating their substantial influence in squash research. Institutional leadership is centered on the English Institute of Sport, the University of Queensland, Monash University, and the University of British Columbia, with the University of Queensland demonstrating an exceptionally high citation impact. Mike Hughes, Ian Franks, and Tim McGarry are recognized as leading figures who account for influential publications.
3. Literature co-citation analysis reveals three distinct research categories: performance analysis, physiological and fitness profiles, and medical and health studies. Performance analysis is the most frequently cited category, while the physiology and fitness cluster shows the strongest internal co-citation relationships. Influential literature emerges from four specific areas: physiological demands, fitness testing, technical and tactical analysis, and epidemiologic studies of squash-related injuries. The absence of recently highly cited studies signals a slowdown in research breakthroughs and underscores the need for renewed innovation.
4. Keywords clustering analysis identifies five research hotspots: performance analysis, physical fitness, neuromuscular, eye injuries, and medical studies, primarily targeting professional players. Research focus has evolved from foundational safety and physiological studies in earlier decades to applied performance enhancement research more recently. Current core research areas include computational modeling, physical fitness and training, biomechanics and equipment, and outcome prediction. Notable research gaps persist in technical and tactical analysis, youth development pathways, sports psychology, and nutrition intervention, particularly for female and youth classes.

This study systematically examined the state of squash research from a bibliometric perspective. The findings construct a comprehensive knowledge framework that underscores the achievements and limitations in the field. As squash makes its historic Olympic debut in Los Angeles in 2028, this research provides a theoretical foundation for evidence-based training optimization and a clear roadmap for future investigations. Future studies should prioritize a more inclusive approach by integrating diverse participant groups, cross-disciplinary methodologies, and advanced technological tools to address current limitations and foster significant innovation and impact in the field.

## Figures and Tables

**Table 1:** Top ten journals by publication volume.

Rank	Source	Publications	Citations	Average Citations	Country	IF/Citescore *	JCR/SJR *
1	Journal of Sports Sciences	14	784	56.00	England	2.3	Q2
2	British Journal of Sports Medicine	12	241	20.08	England	11.6	Q1
3	Journal of Human Movement Studies #	6	34	5.67	England	/	/
4	American Journal of Sports Medicine	4	99	24.75	United States	4.2	Q1
4	Journal of Strength and Conditioning Research	4	88	22.00	United States	2.5	Q2
4	International Journal of Sports Physiology and Performance	4	67	16.75	United States	3.5	Q1
4	Research Quarterly of The American Alliance for Health, Physical Education and Recreation	4	36	9.00	United States	1.4	Q3
4	Physician and Sportsmedicine	4	28	7.00	United States	1.9	Q2
4	European Heart Journal	4	21	5.25	England	37.6	Q1
4	Strength and Conditioning Journal	4	14	3.50	United States	2.2	Q2
4	Journal of Physical Education and Sport	4	7	1.75	Romania	2.8 *	Q3 *

Notes: The journal marked with # indicates that the publication is discontinued. The text marked with * indicates data from Scopus.

**Table 2:** Top 10 countries by publication volume and collaboration.

Rank	Country	Publications	% (of 206)	Citations	Average Citations	No of Collaborating Countries
1	United Kingdom	51	24.76	952	18.67	28
2	Australia	31	15.05	955	30.81	14
3	Canada	21	10.19	688	32.76	4
4	United States	16	7.77	525	32.81	8
5	Qatar	9	4.37	163	18.11	10
5	Germany	9	4.37	104	11.56	4
7	Malaysia	7	3.40	24	3.43	10
7	Slovenia	7	3.40	109	15.57	12
9	France	6	2.91	140	23.33	4
9	Ireland	6	2.91	30	5.00	3
9	Netherlands	6	2.91	57	9.50	7
9	New Zealand	6	2.91	41	6.83	5
9	South Africa	6	2.91	148	24.67	1

**Table 3:** Top 10 institutions by publication volume.

Rank	Institution	Publications	Citations	Average Citations	Country
1	The University of Queensland	8	764	95.50	Australia
1	The University of British Columbia	8	536	67.00	Canada
1	Monash University	8	140	17.50	Australia
1	English Institute of Sport	8	60	7.50	UK
5	Aspire Academy	7	103	14.71	Qatar
5	Sheffield Hallam University	7	88	12.57	UK
7	Northumbria University	6	93	15.50	UK
7	University of Ljubljana	6	74	12.33	Slovenia
9	UNSW Sydney	5	111	22.20	Australia
9	Middlesex University	5	49	9.80	UK
9	The University of Sydney	5	31	6.20	Australia

**Table 4:** Top 10 authors by publication volume.

Rank	Author	Publications	Citations	Average Citations	Country of Affiliation
1	Finch, Caroline	9	159	17.67	Australia/USA
2	Hughes, Mike	8	408	51.00	UK/Ireland
2	Eime, Rochelle	8	140	17.50	Australia
4	Franks, Ian	7	526	75.14	Canada
4	James, Nic	7	85	12.14	UK
6	McGarry, Tim	6	505	84.17	Canada/USA
6	Winter, Edward Mitchell	6	84	14.00	UK
6	Pers, Janez	6	74	12.33	Slovenia
6	Vuckovic, Goran	6	74	13.00	Slovenia
6	Murray, Stafford	6	55	9.17	New Zealand/UK

**Table 5:** Top ten references with the most vigorous citation bursts.

References	Year	Strength	Begin	End	1973–2024
Northcote RJ, 1983, Brit Heart J, V50, P372, [26]	1983	2.99	1984	1988	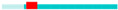
McGarry T, 1995, Hum Perform, V8, P113, [28]	1995	2.54	1996	2000	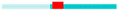
Eime R, 2002, INJ PREV, V8, P239, [29]	2002	2.51	2004	2005	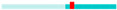
Girard O, 2005, Brit J Sports Med, V39, P921, [25]	2005	3.82	2007	2010	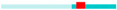
Girard O, 2007, J Strength Cond Res, V21, P909, [24]	2007	3.73	2009	2012	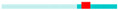
Vuckovic G, 2009, J Sports Sci, V27, P863, [30]	2009	2.63	2013	2014	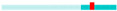
Vuckovic G, 2013, J Sport Sci Med, V12, P66, [31]	2013	2.54	2017	2018	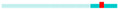
Murray S, 2016, J Sports Sci, V34, P2170, [32]	2016	3.18	2018	2021	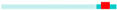
Jones TW, 2018, Int J Sports Sci Coa, V13, P1223, [4]	2018	4.06	2021	2024	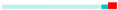
Gibson N, 2019, Strength Cond J, V41, P51, [33]	2019	2.89	2021	2024	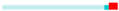

**Table 6:** Top 10 cited literature.

Rank	Reference	Title	Co-Citation Frequency	Cluster Group
1	Girard O, 2007, J Strength Cond Res, [24]	Game analysis and energy requirements of elite squash	27	2
2	Lees A, 2003, J Sports Sci, [11]	Science and the major racket sports: a review	20	2
3	Montpetit RR, 1990, Sports Med, [34]	Applied physiology of squash	18	3
4	Hughes M, 1994, Ergonomics, [22]	Dynamic patterns of movement of squash players of different standards in winning and losing rallies	17	1
5	Girard O, 2005, Brit J Sports Med, [25]	Specific incremental test in elite squash players	15	2
5	Vuckovic G, 2009, J Sports Sci, [30]	Tactical use of the T area in squash by players of differing standard	15	1
7	Sharp NCC, 1998, Sci Racket Sports II, [35]	Physiological demands and fitness for squash	13	2
7	Steininger K, 1987, Brit J Sports Med, [36]	Sports specific fitness testing in squash	13	2
9	Finch C, 2001, Int Sportmed J, [37]	The epidemiology of squash injuries	12	3
10	Vuckovic G, 2013, J Sport Sci Med, [31]	The effect of court location and available time on the tactical shot selection of elite squash players	10	1

## Data Availability

The data that support the findings of this study are available on reasonable request from the corresponding author. The data is not publicly available due to licensing restrictions of the source databases.

## Abbreviations

The following abbreviations are used in this manuscript:

IOC	International Olympic Committee
UK	United Kingdom
USA	United States
AI	artificial intelligence
